# Innate Fear-Induced Weight Regulation in the C57BL/6J Mouse

**DOI:** 10.3389/fnbeh.2016.00132

**Published:** 2016-07-04

**Authors:** Elizabeth A. Genné-Bacon, Joseph R. Trinko, Ralph J. DiLeone

**Affiliations:** ^1^Division of Molecular Psychiatry, Ribicoff Research Facilities, Department of Psychiatry, Yale University School of MedicineNew Haven, CT, USA; ^2^Department of Genetics, Yale University School of MedicineNew Haven, CT, USA

**Keywords:** hypothalamus, weight regulation, mouse models, predator, obesity, ΔFosB, dorsomedial hypothalamic nucleus

## Abstract

Regulation of body weight is an important strategy for small prey animals to avoid capture. Field and laboratory studies have shown that prey animals reduce body size when subjected to long-term predator stimuli. However, the causes of predator-induced weight regulation are highly variable and the underlying mechanisms remain unclear. Understanding this phenomenon is important for gaining a better understanding of how animals regulate body weight under ethologically relevant conditions and has implications for obesity. Here we expose inbred C57BL/6J mice to a fear-inducing odorant (2,4,5-trimethylthiazole; mT) to model predation-induced weight regulation. Eight week-old mice were put on a 45% high fat diet (HFD) or chow diet (5% fat) and exposed daily to mT, an equally aversive dose of butyric acid (BA), or a neutral control scent (almond). mT-exposed mice in both diet groups gained significantly less weight over a 6-week period than BA-exposed mice. This differential weight gain appears unlikely to be due to differences in food intake and activity level, or brown adipose thermogenesis between the mT and BA groups. However, following chronic mT exposure we find increases in ΔFosB protein, a marker for long-term neural plasticity, in the dorsomedial hypothalamus (DMH)—an area previously implicated in chronic stress and defensive responses, as well as weight regulation. This study establishes a simplified and robust laboratory model of predation-mediated weight regulation with inbred lab mice and fear-inducing odor, and suggests a likely, yet undetermined, metabolic adaptation as contributing to this response.

## Introduction

Proper body weight regulation is key to the survival of any animal. This is particularly true for small prey animals, which live under the continuous threat of predation. It is well-documented that prey animals, particularly birds and mammals, reduce body size when predators are present in their environment (Gosler et al., 1995; Lilliendahl, 1997; Carlsen et al., 1999; Gentle and Gosler, 2001; McNamara et al., 2005; Tidhar et al., 2007; Monarca et al., 2015b). This has been hypothesized to be an adaptive response, as smaller animals are able to move faster (Hedenstrom, 1992) and fit into a diverse array of hideaways (Sundell and Norrdahl, 2002), generally making them more difficult prey targets (Lima, 1986; Speakman, 2007). However, the mechanisms underlying this weight response are unclear.

Both field and laboratory studies have produced mixed results on the behavioral and physiological mechanisms responsible for changes in weight after predator exposure. For example, field research (Sundell et al., 2004) has been inconsistent on the role of reduced foraging and food intake in contributing to weight loss (Lima and Bednekoff, 1999). This uncertainty may be due to the challenges of field methodology where many factors remain uncontrolled. Laboratory studies allow for better control of conditions, but have also yielded mixed results on the mechanisms underlying weight changes. For example, a laboratory study of predator scent exposure in field voles found a significant reduction of weight in predator-exposed animals compared to control animals, but no difference in food intake between groups (Carlsen et al., 1999). In contrast, Monarca’s studies of predatory bird calls in wood mice (Monarca et al., 2015b) and C57BL/6J laboratory mice (Monarca et al., 2015a) found that weight differences were likely driven by alterations in food intake. Additionally, Tidhar’s study of weasel feces exposure in bank voles found increased daily energy expenditure associated with reduced body weight, but variability in food intake differences (Tidhar et al., 2007). While these studies have advanced our understanding of predation-mediated weight regulation, there is still great need to standardize protocols to resolve discrepancies and better define the mechanisms involved.

Choice of predator stimulus is likely to influence the mechanisms underlying predator-mediated weight regulation. Scent is a particularly salient sense in rodents (Doty, 2012), and therefore most studies use the natural scent of predator feces, urine, or fur as a stimulus. While this strategy closely imitates conditions prey animals might encounter in the wild, it is difficult to fully control and replicate these natural scents between studies and subjects, making this approach somewhat limiting. Modeling predator stimulus-mediated weight regulation with an easily controlled and replicable predator-like scent could help clarify mechanisms and compliment the work already done with other stimuli. 2,4,5-trimethylthiazole (mT) is a chemical structurally very similar to the red-fox derived predator scent 2,4,5-trimethylthiazoline (TMT; Brechbühl et al., 2013a; see “Materials and Methods” Section), and produces several innate fear responses such as avoidance (Brechbühl et al., 2013a) and freezing (Kobayakawa and Kobayakawa, 2011). Both TMT and mT are synthetic compounds, making them easily quantified and controlled, greatly reducing problems with variability encountered with natural predator stimuli.

Selection of animal model is also essential for reducing variation and standardizing protocols for studying predator responses. The C57BL/6J mouse is a commonly used laboratory mouse strain that offers an extensive genetic toolkit for more detailed neural studies of mechanisms that influence predator responses. Despite separation from wild conditions, it is known that C57BL/6J mice do respond to a variety of predator stimuli (e.g., Cohen et al., 2008; Janitzky et al., 2015; Monarca et al., 2015a). Additionally, C57BL/6J mice gain weight on a high-fat diet, and are commonly used as a model for obesity and body weight regulation. Because of this, establishing the C57BL/6J mouse as a model for predator-mediated weight regulation allows us to bridge knowledge gained from ecological studies with biomedical studies of weight regulation.

Defining mechanisms governing predator-mediated weight regulation are of both ecological and biomedical relevance. Understanding the neurobiological basis of predator responses will help to inform our view of predator-prey interactions in wild animals. Additionally, defining physiological and behavioral changes that regulate body weight under ethologically relevant conditions may improve our understanding of the etiology of obesity in these important model animals.

## Materials and Methods

### Animals

All animals used were inbred male C57BL/6J and purchased from The Jackson Laboratory (Bar Harbor, ME, USA) between 7 and 8 weeks of age. Mice were group housed (five or three to a cage, except where indicated otherwise) in standard mouse cages, kept on a 12-h light/dark cycle (lights off at 7 pm), and provided *ad libitum* access to food and water. Mice were allowed to acclimate to our facility for at least 1 week prior to any manipulations and were between 8 and 10 weeks of age at the onset of experimentation. All experiments were carried out in accordance with Yale University Institutional Animal Care and Use Committee (IACUC) protocol.

### Scents and Controls

#### Note on TMT and mT

There are several distinct chemicals commonly referred to as “fox odor” in the literature, including: the *cis-* and *trans-* isomers of TMT (e.g., Vernet-Maury, 1980; Wallace and Rosen, 2000; Endres et al., 2005); 2,5-dihydro-2,4,5-trimethylthiazole, an isomer of the first chemicals, differing in placement of the ring double bond, (e.g., Vernet-Maury et al., 1984; Ihara et al., 2013); and mT, an unsaturated variant of the first two chemicals (e.g., Mueller and Bale, 2008; Howerton and Bale, 2014). However, only the TMTs have been directly found in the secretions of the red fox, a natural predator of rodents (Vernet-Maury, 1980). Nonetheless, other researchers have sought to improve the predator fear-like response to fox odor by creating a variety of thiazole/ine-related fear odors, which produce similar or greater degrees of threat responses (Kobayakawa and Kobayakawa, 2011; Isosaka et al., 2015). mT was shown to produce similar levels of freezing compared to TMT (Kobayakawa and Kobayakawa, 2011). mT also activates the Grueneberg ganglion (Brechbühl et al., 2013a), an olfactory sub-system which is important for responses to alarm pheromones and predator scents, and required for TMT-induced freezing (Brechbühl et al., 2013b). Thus, though mT is itself not a predator odor, it is an innate fear-inducing odor, eliciting many of the behavioral and physiological responses of TMT.

The fear-inducing odor mT (Sigma Aldrich, 98%) was used as a model of predator fear. Mice were exposed to all scents by pipetting liquid odorant onto a small square of KimWipe in a small (4.6 × 4.6 cm) weigh boat and placing it in a standard mouse cage (or other apparatus where described) with the mice. Diluted concentrations of mT (see“Center Avoidance Test” Section below) were made by creating an emulsion with distilled water. Undiluted butyric acid (“BA”, ≥99%, Sigma-Aldrich, St. Louis, MO, USA), or almond scent (Badia Spices, Doral, FL, USA) were used for controls.

### Center Avoidance Test

**Figure 1:**
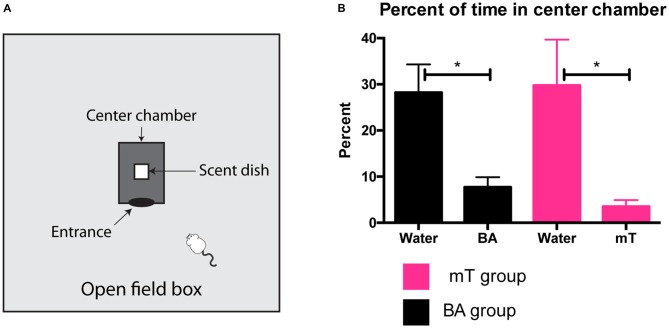
**Establishing an aversive control for 2,4,5-trimethylthiazole (mT).** Four groups of mice (*n* = 5 per group) were habituated to an open field containing an opaque center chamber where either water or different quantities of mT and butyric acid (BA) were presented **(A)**. Behavior was recorded over 10 min sessions and percent of time spent in the center chamber was compared between water and scent presentation. 52.8 μl of BA and 9.8 μl of 50% mT produced approximately equal levels of avoidance **(B)**. All error bars shown represent standard error of the mean (SEM).

Four cages of group-housed mice (five per cage) were used to determine approximately equivalently aversive doses of mT and BA. Mice were first habituated to the testing apparatus for 2 days of 10-min sessions. The testing apparatus consisted of an open field box containing a smaller opaque box within the center of the field (the “center chamber” Figure 1A). Mice had access to the center chamber through a single opening. On the 3rd day water was presented in the center chamber for all cages. On the 4th day each cage of mice was presented with one of four possible scents/concentrations: 9.8 μl mT, 9.8 μl mT diluted 1:1 in distilled water, 52.8 μl BA, and 105.6 μl BA. Time spent in the center chamber on 3rd and 4th days was recorded and scored in real time using the behavioral software AnyMaze (Stoelting Co., Wood Dale, IL, USA).

### Chronic Scent Exposure Test

To model predator-mediated weight regulation, we examined weight gain during daily mT scent exposure. Beginning on experiment day 0, each cage of mice was taken to a separate room, put in a fresh standard cage with bedding, and presented with either 30 μl almond scent, 52.8 μl BA, or 9.8 μl 50% mT for 30 min. Scent exposures were repeated at approximately the same time each day (early afternoon—light period). After first scent exposure, high fat diet (HFD)-assigned animals were switched from chow (5% Kcal from fat, RMH-3000, LabDiet, St. Louis, MO, USA) to a 45% HFD (D12451, Research Diets, New Brunswick, NJ, USA). Mice were weighed every 3 days for 6 weeks. Weight data from chow-fed mT and BA groups were lost for experiment day 10 and so are not shown. Due to space limitations, the almond exposures were performed on a separate cohort from the mT and BA exposures. Conditions were kept the same between cohorts.

### Food Intake Measurements

To measure food intake, the chronic scent exposure experiment (described above) of mT and BA was repeated using five cages per experimental group of paired-housed mice (20 mice total) fed a HFD. Body weight and food intake were recorded daily for a period of 6 weeks. Food intake was assessed by subtracting the amount of food remaining in the cages from the amount provided to the animals the previous day. Food spillage was minimal, and was assessed by visual inspection and accounted for when necessary.

### Locomotor Activity Measurements

To measure locomotor activity, one mouse from each cage from the above food intake experiment cohort was removed from its home cage after 21 days of scent exposure and moved to individual cages containing food, water, and bedding, and placed in locomotor boxes (Med Associates, St Albans, VT, USA) at the onset of the night cycle. Locomotor activity counts were defined as consecutive beam breaks, and recorded using Med-PC IV software for a period of 22 h.

To study locomotor activity in more detail, the chronic exposure protocol was again repeated, using group-housed mice (five mice per cage, one cage per scent group). To obtain a baseline reading prior to beginning of scent exposure and HFD, mice were placed in locomotor boxes (as described above) at approximately 3 pm and removed 22 h later. Following the start of daily scent exposure, locomotor activity was reassessed weekly for a period of 4 weeks. On the days of locomotor activity testing, scent exposure was conducted during the 2-h period prior to the beginning of locomotor testing. Locomotor boxes were located in a separate room.

### Corticosterone Measurements

#### Acute Exposure

Group housed mice (five per cage) were habituated to the scent exposure setup for 2 days prior to sacrifice. On day of sacrifice, animals were exposed to 52.8 μl BA, 9.8 μl 50% mT, or 30 μl almond scent for 30 min, and then sacrificed by rapid decapitation and trunk blood was collected. Scent exposure and sacrifice was performed on separate days for different scent groups, at the same time each day (early afternoon). Two hundred fifty microliter of trunk blood was immediately added to 100 μl of 2% EDTA (Sigma-Aldrich) and spun for 15 min at 2000 g at room temperature; plasma was collected as the supernatant.

#### Chronic Exposure

Animals from a separate cohort (*n* = 6 per scent group, two cages of three mice each per group) of the chronic scent exposure experiment were sacrificed by rapid decapitation following the final scent exposure session (21 scent exposures total). Trunk blood was collected and processed as described above.

### Plasma CORT Measurements

Plasma corticosterone (CORT) was measured with Assay Designs ELISA kits according to manufacturer’s protocol, as described previously (Guarnieri et al., 2012). Briefly, 1 μl of diluted plasma (1:50), was compared to known concentrations. An OD reading at 405 nm with correction at 570 nm was taken, a standard curve was generated, and unknowns were extrapolated using the Prism statistical software (GraphPad Software, La Jolla, CA, USA).

### Temperature Measurements

To evaluate changes in body temperature, mice from the locomotor activity cohort (see above) were briefly anesthetized with isoflurane and implanted with IPTT-300 temperature transponders (Bio Medic Data Systems, Seaford, DE, USA). Temperatures were recorded with a non-invasive probe (DAS-5007 IPTT pocket scanner, Bio Medic Data Systems) prior to daily scent exposure.

#### Brown Adipose Tissue Dissection and qPCR

To search for evidence of metabolic changes, uncoupling protein 1 (*Ucp1*) mRNA from brown adipose tissues (BAT) was measured in both chronic and acute scent-exposed mice. Chronic-exposure mice were sacrificed on the 36th day of scent exposure. Acute exposure tissue collection timing (5 h post-scent) was based on peak *Ucp1* mRNA expression timing from cold challenge studies (Nedergaard and Cannon, 2013). Following sacrifice by rapid decapitation, whole interscapular BAT was collected and quickly frozen on dry ice and stored at −80°C. RNA samples were purified from approximately 0.1 g of frozen BAT using Trizol reagent (Invitrogen, Carlsbad, CA, USA); a Nanodrop ND-100 (Thermo Fisher Scientific, Waltham, MA, USA) was used to quantify purified total RNA. cDNA samples were made from 1000 ng RNA using Superscript III Reverse Transcriptase kit and qPCR was carried out using Taqman gene expression kits. The 2^−ΔΔCt^ method was used for calculating fold change compared to TATA-binding protein (*Tbp*) control (Livak, 2001). Primer kits used:

— Mm01244861_m1 *Ucp1* TaqMan^®^ Gene Expression Assay (Thermo Fisher).
— Mm01277042_m1 *Tbp* TaqMan^®^ Gene Expression Assay (Thermo Fisher).

#### Brain Dissection and ΔFosB Measurement

To identify brain regions that may be involved in threat response, ΔFosB, a marker of neural plasticity, was measured after chronic scent exposure. Brains were collected after rapid decapitation and quickly frozen and stored at −80°C. Brains were then briefly partially thawed and then sectioned using a 1 mm brain block (BrainTree Scientific, Braintree, MA, USA) and each region was microdissected with guidance from the Mouse Brain Atlas (Paxinos and Franklin, 2004) using a scalpel, except for the nucleus accumbens (NAc) and Amygdala, where a 12 gauge circular punch was used. Protein extraction and analysis was carried out as described previously (Sears et al., 2010). Briefly, frozen tissue samples were sonicated and then boiled for 20 min in a 1% SDS lysis buffer solution containing 1:100 protease inhibitor (Sigma P8340) and 1:100 phosphatase inhibitors (Sigma P5726 and P0044). Total protein concentration was assessed with a Pierce bicinchoninic acid (BCA) protein assay (Thermo Fisher). One sample (BA#1) from the medial hypothalamus (mHyp) had insufficient protein concentration and was excluded from further analysis. Equal quantities of protein (55 μg) were separated by SDS polyacrylamide gel electrophoresis (Bio-Rad mini-PROTEAN TGX precast 12% polyacrylamide gels), and transferred to nitrocellulose membranes (0.2 μm, Bio-Rad, Hercules, CA, USA). The membranes were immunoblotted using a 1:1000 dilution rabbit anti-FosB (Cell Signaling Technology, Danvers, MA, USA) and a 1:2000 dilution of mouse anti-β-actin (Cell Signaling). Antibody binding to FosB and ΔFosB was visualized by incubation with a 1:10,000 dilution of donkey anti-rabbit horseradish peroxidase-linked IgG (Vector Laboratories, Burlingame, CA, USA) and developed using Western Lightning Plus ECL chemiluminescence HRP substrate (PerkinElmer, Waltham, MA, USA) and a ChemiDoc imager (Bio-Rad). Antibody binding to β-actin was revealed using the LI-COR Odyssey quantitative infrared western blot detection system (IRDye 680LT secondary antibody, LI-COR, Lincoln, NE, USA). Band densities were analyzed using the Gel Analysis tool in ImageJ.

#### FosB Immunofluorescence and Cell Counting

Immunoflourescent labeling was used to visualize FosB/ΔFosB protein expression in hypothalamic nuclei. Chow fed mice were exposed to 36 days of daily scent exposure (six mice per scent group) as described above. Ninety minutes following the final scent exposure animals were sacrificed through intracardial perfusion of 10% formalin, and brains were collected, processed, and cut to 40 μm sections as described previously (Land et al., 2014). Staining for FosB (rabbit-anti-FosB, Cell Signaling; 1:500) and secondary antibody (donkey-anti-rabbit Alexa 555; 1:500) was done in 3% normal donkey serum and 0.3% Triton X-100. Tissue was visualized and images were captured using a fluorescent microscope, as described previously (Land et al., 2014). Images were taken at 10× magnification, with three 40 μm sections quantified per animal. FosB labeling was quantified using ImageJ on matched sections by a researcher blinded to the experimental groups.

#### Statistics and Analyses

All statistical analyses were carried out using the Graph Pad Prism software package (version 6). Unpaired two-tailed *t*-tests were used for comparisons between two groups. One-way ANOVA was used for comparison between more than two groups. Two-way repeated measures ANOVAs were used for comparisons of two or more groups over time. Tukey corrections for multiple comparisons were used to test for significance among individual groups within ANOVAs. Significance threshold (alpha) was set at 0.05 for most tests with levels of significance defined as follows: *p ≤ 0.05, **p ≤ 0.01, ***p ≤ 0.001. For ΔFosB measurements in the brain, the Benjamini–Hochberg procedure (with a false discovery rate set at *Q* = 0.2) was used to correct for multiple testing (adjusted alpha = 0.0286).

## Results

### Establishing an Aversive Control for mT

Both mT and TMT, in addition to being innate fear-inducing odors, are also aversive and noxious scents. BA is commonly used as a control for TMT due to its strong non-predator associated noxious odor (e.g., Morrow et al., 2000; Endres et al., 2005). In order to determine doses of mT and BA that were similarly aversive, we set up a test of avoidance of a center chamber inside an open field. Four separate groups of mice were exposed to different scent quantities (see Figure 1A), and time spent in the center chamber was scored and compared to a no-scent control session. We found 52.8 μl of undiluted BA produced approximately equivalent levels of avoidance to 9.8 μl of a 50% mT dilution (see Figure 1B). To examine the fear-inducing potential of these quantities of BA and mT, we conducted a test for freezing (a common behavioral threat response; see Supplementary Methods). Although these quantities of BA and mT produce equal aversive behavior, we found mT significantly elevated freezing (a threat response) compared to BA, as well as to no-scent (water) and neutral scent controls (*p* < 0.0001). BA did not significantly elevate freezing compared to no-scent or neutral scent control (see Supplementary Figure 1). These quantities were used in all subsequent scent exposure experiments.

### Chronic mT Exposure Attenuates Weight Gain in Both Low and High Fat Diets

**Figure 2:**
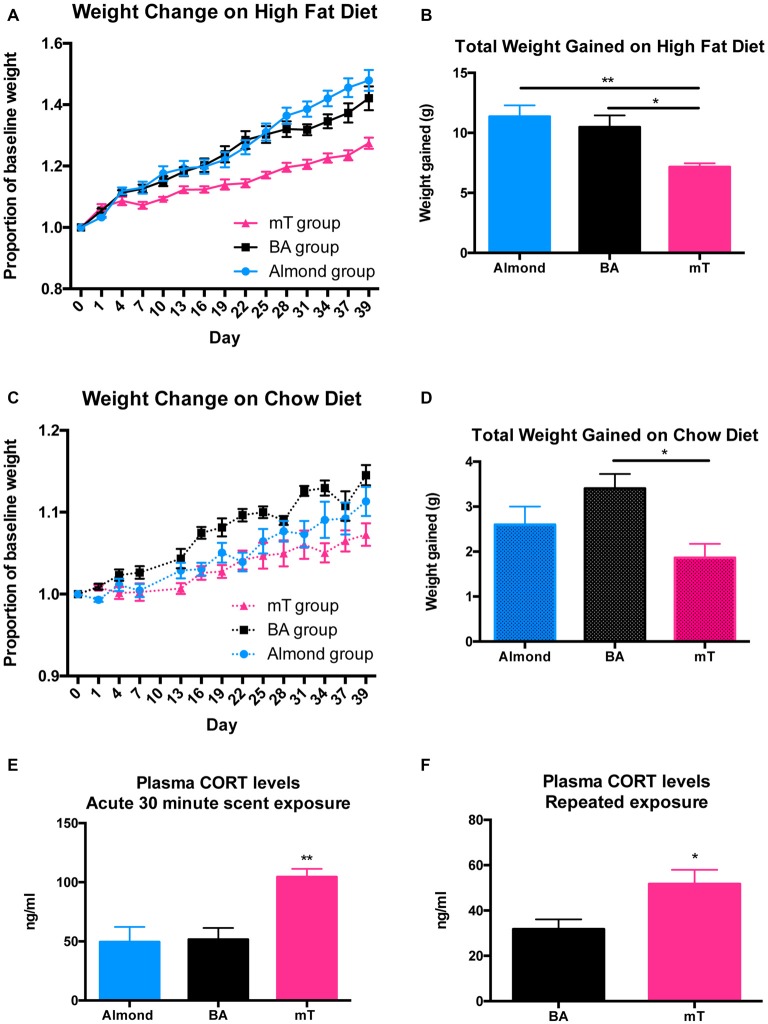
**Chronic mT exposure attenuates weight gain in both low and high fat diets (HFDs).** Six cages of group housed (*n* = 5 per cage) were exposed daily to 9.8 μl mT, 52.8 μl BA, or 30 μl almond scent for 6 weeks while on a chow (5% fat) or high fat (45% fat, HFD) diet. **(A)** Weight gain on HFD presented as fold change from baseline weight. **(B)** Total weight gained on HFD at the end of the 6-week experiment. mT-exposed mice gained significantly less weight than BA or Almond exposed groups. **(C)** Weight gain on chow diet presented as fold change from baseline weight. **(D)** Total weight gained on chow diet. mT-exposed mice gained significantly less weight than BA-exposed mice on a chow diet. **(E)** Plasma corticosterone (CORT) levels from trunk blood after a single 30-min exposure to 9.8 μl mT, 52.8 μl BA, or 30 μl almond scent. **(F)** Plasma CORT levels from trunk blood after 3 weeks of daily scent exposure (and immediately after the final 30 min scent exposure). All error bars shown represent SEM.

To determine if mT exposure will affect weight gain in C57BL/6J mice, six cages of group-housed mice (*n* = 5 per group) were exposed daily to mT, BA, or almond scent and given *ad libitum* access to either a 45% HFD or a standard chow (5% fat) diet. For the HFD-fed groups, there was a significant effect of scent (*p* = 0.0024, *F* = 10.43; 2-way ANOVA), day (*p* < 0.0001, *F* = 366.8), and a significant interaction (*p* < 0.0001, *F* = 15.27). Animals exposed daily to mT gained significantly less weight over time than mice exposed to almond scent control (*p* < 0.05) or equally-aversive BA control (*p* < 0.01; see Figures 2A,B). The BA-exposed group was not significantly different from the almond scent control in the HFD fed group. In the chow-fed group, differences were less robust. While a 2-way ANOVA revealed a significant main effect of scent (*p* = 0.0212, *F* = 5.401), as well as a significant interaction between scent and day (*p* < 0.0001, *F* = 4.290), testing for differences between individual scent groups revealed only significant differences between BA and mT groups (*p* < 0.05), while the almond group did not significantly differ from either Figure 2C. Total weight gained over the 6-week period was significantly lower in the mT exposed animals compared to BA-exposed animals in both diet groups (Figure 2D), with BA-exposed mice gaining an average of 3.32 g (±1.023 g) more than mT-exposed mice over the 6 week testing period for the HFD-fed group and 1.54 g (±0.4512 g) for the chow-fed group. As differences in weight gain between scent groups were more robust in HFD animals, we chose to focus primarily on this diet group for further experiments.

### mT Elevates Plasma Corticosterone After Acute and Repeated Exposures

To determine approximate stress levels provoked by mT and BA, we measured plasma CORT, a common marker of stress in rodents, after acute (one time) and repeated scent exposure (21 days of daily exposure). Plasma CORT was significantly elevated (*p* = 0.0026, *F* = 10.74; 1-way ANOVA) following a single exposure to 9.8 μl 50% mT compared to 52.8 μl BA or 30 μl almond scent (BA = 51.62 ng/ml, Almond = 49.48 ng/ml, mT = 104.46 ng/ml). Plasma CORT levels did not differ between BA and almond scent exposures, suggesting differential stress responses despite equivalent aversion levels (See Figure 2E). Plasma CORT levels after repeated daily exposure to mT (Figure 2F) were also elevated compared to BA (*p* = 0.0248, BA = 24.08 ng/ml, mT = 39.45 ng/ml), however plasma CORT levels following repeated mT exposure were significantly decreased (*p* = 0.0007, mean difference of 35.16 ± 7.01 ng/ml comparing Figures 2E,F) compared to after only a single exposure.

### Chronic mT does not Significantly Change Food Intake

**Figure 3:**
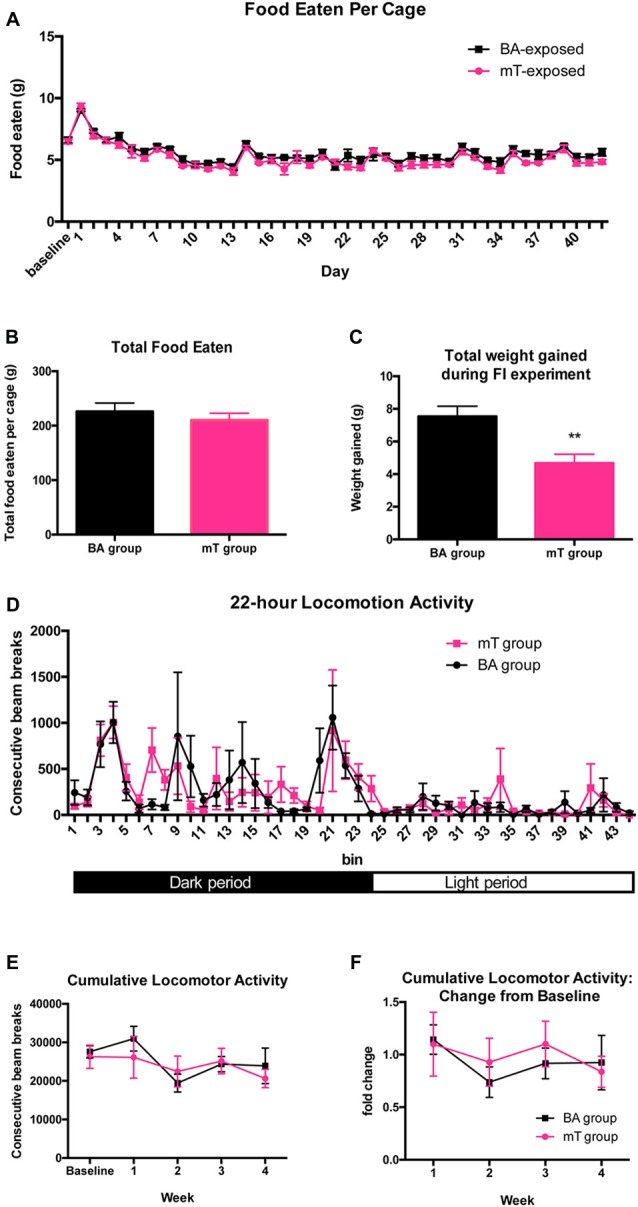
**Weight attenuation following chronic mT exposure is not explained by differences in food intake or locomotor activity. (A)** Daily food intake per cage (two mice per cage, five cages per group) of mice on HFD and experiencing daily scent exposure. **(B)** Total food intake per group over the 6-week experiment. **(C)** No differences were seen in food intake despite a significant attenuation of weight gain in mT-exposed mice. **(D)** Locomotor activity for mice undergoing daily scent exposure. Mice were placed in new locomotor boxes with bedding at the onset of the night cycle (~5 h after daily scent exposure). Locomotor counts measured as two consecutive beam breaks. Black and white bar represents night/day cycle. **(E)** Cumulative locomotor activity counts over 22-h periods for a new cohort of chronic scent-exposed mice tested for locomotor activity weekly. **(F)** Cumulative locomotor counts divided by a pre-scent baseline day. All error bars shown represent SEM.

To determine if the difference in weight gain between mT and BA-exposed mice could be driven by differences in food intake, an independent cohort of animals housed two to a cage (*n* = 5 cages per experimental group and *n* = 10 animals per group total) was put on 45% HFD and exposed daily to mT or BA for a period of 6 weeks with food intake and body weight measured daily. As expected, mT-exposed animals gained significantly less weight than BA-exposed animals when put on HFD (2-tailed *t-test*: *p* = 0.0028, Figure 3C). However, there was no significant difference between groups for food intake over time (scent: *p* = 0.1179, *F* = 3.068; 2-way ANOVA, see Figure 3A) or total food eaten between groups (*p* = 0.1148, 2-tailed *t* test; see Figure 3B).

### Chronic mT Exposure does not Affect Locomotor Activity

To determine if changes in physical activity could be driving differences in weight gain, this cohort of animals was also tested for locomotor activity over a 22-h period. Following daily scent exposure, one mouse from each of the double-housed cages was placed in a locomotor box and activity was recorded over a 22-h period. No differences in the pattern of locomotion (as measured by consecutive beam breaks) were detected between groups (Figure 3D). To further assess potential differences in physical activity, an independent cohort was subjected to the chronic scent exposure test. Locomotor activity in these mice was recorded for 22-h periods once per week, including a baseline reading prior to any scent exposure. Again, no differences in the hourly circadian rhythm of locomotor activity was observed between groups, nor were changes observed over time (Figures 3E,F), despite a significant difference in weight gain between groups (BA: 6.86 g gained, mT: 3.6 g gained; *p* = 0.0008, 2-tailed *t*-test, see Supplementary Figure 2).

### Chronic mT does not Change Body Temperature or Uncoupling Protein mRNA Levels

**Figure 4:**
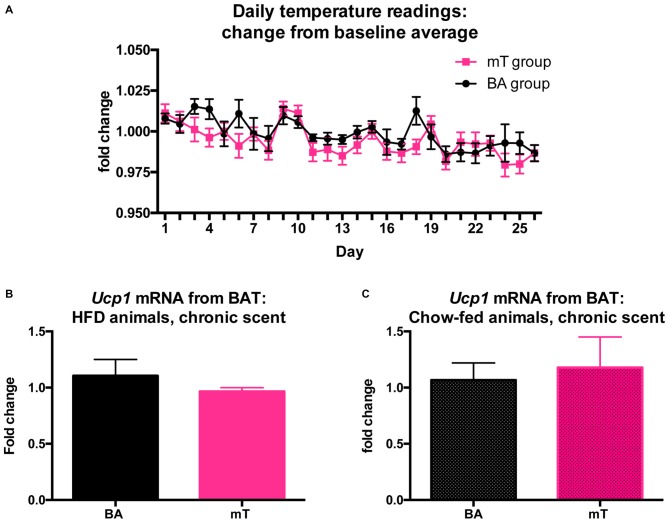
**No evidence for changes in thermogenesis after mT exposure. (A)** Daily temperature readings relative to pre-scent baseline period. Mice were implanted with subdermal temperature transponders and exposed to daily scents while on a HFD. No main effect of scent was observed. Uncoupling protein 1 (*Ucp1*) mRNA levels measured with qPCR from brown adipose tissue (BAT) for HFD **(B)** and chow **(C)** fed animals exposed to 36 days of daily scent. Fold change compared with TATA-binding protein (*Tbp*) and calculated using the 2^−ΔΔCt^ method. All error bars shown represent SEM.

To investigate a potential thermogenic effect of stress contributing to differences in weight gain, we recorded daily temperatures in chronic mT exposed animals. Transponders were inserted subdermally between the scapulae and no main effect of mT on temperature was detected (*p* = 0.4495, *F* = 0.6322; Figure 4A). We also tested a molecular marker of thermogenesis: UCP1 mRNA from BAT using qPCR. A new chronic scent exposure cohort (with both HFD and chow-fed groups, *n* = 6 per treatment/food group) was sacrificed and BAT collected from each animal after 36 days of scent exposure. No differences in *Ucp1* mRNA level were detected between scent groups in either HFD (Figure 4B) or chow (Figure 4C) fed groups. We additionally measured *Ucp1* mRNA from BAT following an acute (one-time) scent exposure in naive animals, but again did not see any changes in *Ucp1* mRNA expression between scent groups (See Supplementary Figure 3).

### Changes in a Marker of Neural Plasticity Following Repeated mT Exposure

**Figure 5:**
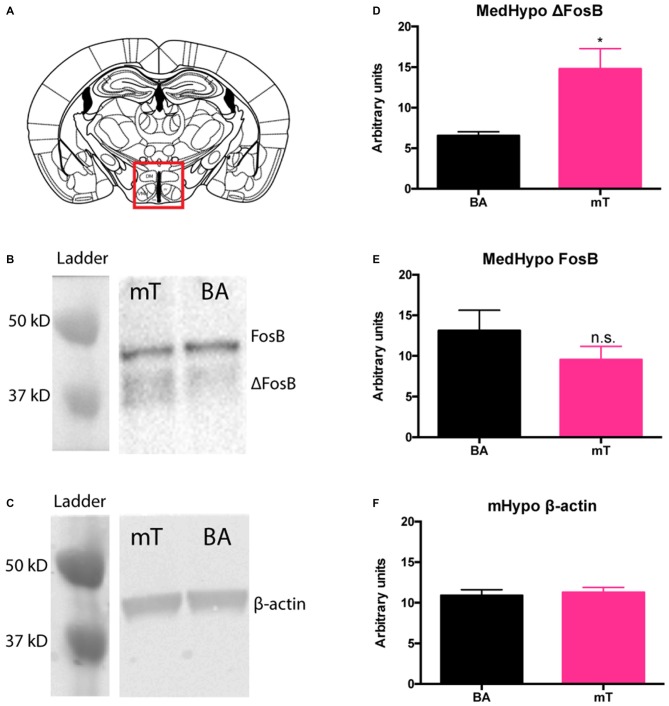
**ΔFosB levels are increased in the medial hypothalamus (mHyp) following repeated mT exposure.** Western blots for FosB and β-actin (loading control) were carried out on fresh-frozen brain tissue dissected from the mHyp **(A)**. Protein bands were distinguished by size **(B,C)**. Band intensities were calculated using ImageJ gel analysis tools. ΔFosB protein levels were significantly elevated **(D)** in the mHyp following 6 weeks of daily mT exposure compared to BA-exposed mice. Neither FosB levels **(E)**, nor β-actin** (F)** differed between scent groups. All error bars shown represent SEM.

To investigate which regions of the brain may be involved in the response to chronic mT exposure, we measured ΔFosB, a marker of long-term neural plasticity, in a survey of several brain regions previously implicated in TMT and other predator threat responses. Following 6 weeks of mT or BA scent exposure (*n* = 5 per scent group), chow-fed mice were sacrificed and brains sectioned in a brain block to 1 mm slices. Brain tissue from the septal nucleus (LS), NAc (core and shell), bed nucleus of the stria terminalis (BNST), anterior hypothalamus (AHN), mHyp (encompassing both the dorsomedial and ventromedial hypothalamic nuclei), amygdala (basolateral), and whole hippocampus was dissected (Supplementary Figure 4), isolated, and FosB and ΔFosB protein levels measured with western blot. We found significant elevation of ΔFosB protein between mT and BA exposed mice in the mHyp (*p* = 0.024, 2-tailed *t test*), despite no change in total FosB (*p* = 0.256) or β-actin (*p* = 0.692; Figure 5). No differences in FosB or ΔFosB protein levels were detected in any other brain region.

**Figure 6:**
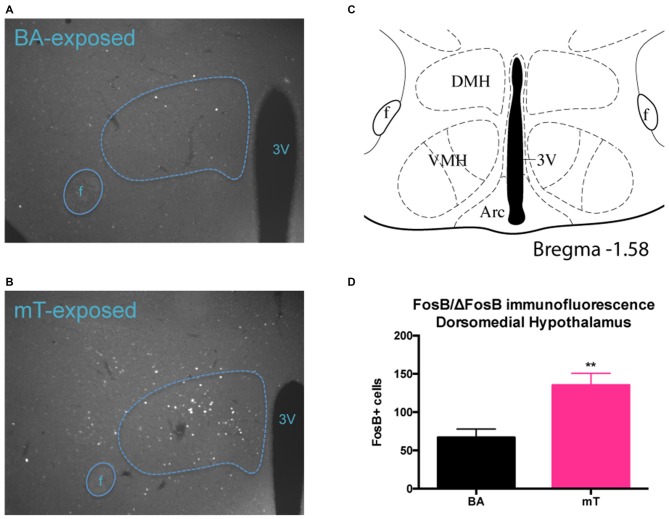
**FosB/ΔFosB levels are increased specifically in the dorsomedial hypothalamus (DMH) following repeated mT exposure.** FosB/ΔFosB immunofluorescent staining was carried out on perfused brain tissue from chronic scent-exposed mice. **(A,B)** Representative images of FosB/ΔFosB expression in the DMH in a BA or mT-exposed mouse brain, respectively. Dotted line represents DMH based on approximate brain region boundaries **(C)** from Paxinos and Franklin (2004); 3V, third ventricle, f, fornix, VMH, ventromedial hypothalamus, Arc, arcuate nucleus. Significantly more FosB/ΔFosB positive cells were counted in the DMH of chronically mT-exposed mice, compared to BA-exposed **(D)**. Error bars shown represent SEM.

To visualize which fine structure(s) in the mHyp may be contributing to differential ΔFosB protein expression, immunofluorescent staining of total FosB/ΔFosB protein in the mHyp was carried out on perfused brain tissue from chronically scent exposed, chow fed mice. We found a significantly higher number of FosB/ΔFosB positive cells in the dorsomedial hypothalamus (DMH) in mT-exposed mice (2-tailed *t*-test, *p* = 0.0047, see Figure 6). Very little, if any, FosB/ΔFosB expression was observed in the ventromedial hypothalamus (VMH) in either scent group.

## Discussion

In this study we find significant attenuation of weight gain in C57BL/6J mice on both a high fat and low fat diet when exposed daily to a threatening scent compared to a non-threatening, but equally aversive, scent. We find no evidence for the involvement of differences in food intake, locomotor activity, or brown adipose thermogenesis in mediating this differential weight gain. We do find significant increases in a marker of neural plasticity (ΔFosB) in the mHyp, a brain region thought to be involved in processing predator threat (Canteras, 2002). This study provides additional evidence for the role of predation stimuli in regulation of body weight in rodents, and demonstrates that predation-mediated weight reduction can be modeled using inbred laboratory mice and a synthetic fear-inducing olfactory stimulus.

We find that mT-exposure attenuates weight gain in mice on both high and low fat diets, with HFD showing more robust and consistent changes. This is likely due to the relatively small overall weight gain seen in each scent group for the chow fed animals. HFD provokes a larger change in weight, making the differences between scent groups more distinct. Additionally this study uses only relatively young, male mice, and different responses may be seen in different age groups or sexes.

Previous studies have implicated alterations in food intake and physical activity to explain predator-mediated weight regulation (Tidhar et al., 2007; Monarca et al., 2015a,b). However, we find no evidence to support the contribution of these behaviors to the difference in weight with mT exposure. It is possible for the differences we see in weight gain to be due to very small changes in food intake that we lack the power to detect. However, our results are in contrast to a similar study with auditory cues, where changes in weight of C57BL/6J mice were clearly driven by differences in food intake (Monarca et al., 2015a). This conflict is not without precedent, as other studies have found no difference or mixed differences in food intake or foraging behavior with exposure to predator stimuli (Carlsen et al., 1999; Sundell et al., 2004). It is likely that these discrepancies are due to differences between experimental protocol, species used, or chosen stimulus. For example, diurnal timing of scent exposure may affect factors such as food intake, and presenting scent during the animals’ natural feeding period could result in greater changes in food intake than observed here. Further studies controlling for each of these variables of predator stimulus presentation will help to tease apart the complex factors that encompass predator threat response.

This study uses mT, a fear-inducing odor structurally very similar to the predator odor TMT, which is found in the excretions of red fox anal glands (Vernet-Maury, 1980; Brechbühl et al., 2013a). We are able to recapitulate several previously observed components of predator-mediated weight regulation (e.g., reduced body weight, increases in ΔFosB in the mHyp), while others (e.g., food intake changes) are absent. It is possible that mT is not a close enough mimic of predator stimuli, and further studies should be done to directly compare mT with TMT and with other predator odors. There is also evidence that single-molecule odorants, such as TMT, do not elicit the full range of predator threat responses in rodents (Staples et al., 2008; Pagani and Rosen, 2009). It is possible that we are observing a very specific component of the complete predator scent response repertoire and other responses require different stimuli. Several other single-molecule predator-associated odorants have now been discovered (Rosen et al., 2015), and these may elicit other predator-threat responses seen in response to natural predator odors but not to TMT or mT.

The underlying mechanism for the mT-provoked weight response seen in our animals remains unclear. Because there were no detectable changes observed in food intake or physical activity, metabolic changes are suspected. Metabolic changes have been previously implicated in weight response to chronic stress (Michel et al., 2005; Harris, 2015), as well as specifically to predator-mediated weight regulation (Tidhar et al., 2007). We find equally aversive fear-inducing odor and non-fear inducing control odors elicit different CORT responses, with mT-exposed mice showing elevated CORT levels both acutely and chronically, indicating that our mT-exposed animals are under chronic stress (Tidhar et al., 2007).

Stress-induced hyperthermia has been proposed as the primary metabolic component of stress-induced weight loss (Arase et al., 1988). BAT is the body’s primary source of non-shivering thermogenesis and known to be activated by certain kinds of stress (Ricquier and Mory, 1984; Cannon and Nedergaard, 2004). Because of this, we measured expression of the primary heat-producing molecule of BAT, UCP1, from intrascapular BAT through qPCR of *Ucp1* mRNA in chronically mT-exposed mice. We found no differences in BAT *Ucp1* mRNA expression between scent groups after repeated mT exposure. Though mRNA expression is only a proxy-measurement for the thermogenic capacity of BAT (Nedergaard and Cannon, 2013), we also did not see corresponding changes in body temperature from intrascapularly placed temperature transponders, leading us to conclude that local thermogenesis is likely not a major contributor to mT-induced weight attenuation.

This study finds increases in expression of ΔFosB protein, a persistent transcription factor involved in long-term regulation of gene expression, in the mHyp following 6 weeks of daily exposure to a fear-inducing, predator-like stimulus. This is consistent with increases in *FosB/ΔFosB* mRNA seen in the mHyp of Bradt’s voles after repeated exposure to cat feces, as well as changes in FosB/ΔFosB immunoreactivity in the VMH (dorsomedial region-VMHdm) of rats after repeated exposure to cat fur (Staples et al., 2009; Hegab et al., 2014). To our knowledge, our study is the first to confirm it is ΔFosB protein, a truncated isoform of FosB known to mediate long-term neural plasticity, that is elevated in this region, as no differences were detected for FosB protein between scent groups. Additionally, the comparatively long time course used for our study indicates that this region is highly resistant to habituation and/or continues to be important to long-term threat responses.

Interestingly, previous evidence for the mHyp’s role specifically in TMT-response (as opposed to other predator odors) has been mostly negative. While the role of the VMHdm in response to natural predator odors such as cat fur is well established (Canteras, 2002), multiple studies show no immediate early gene activation in the VMHdm following TMT exposure (Staples et al., 2008; Pérez-Gómez et al., 2015). Similarly, we observed low expression of FosB/ΔFosB in the VMH (including the VMHdm) of chronically mT-exposed mice, but do see a robust increase in FosB/ΔFosB in the DMH in mT-exposed mice. Since protein expression from our western blot shows no differences in FosB in the mHyp, the increased number of FosB+ immunofluorescent cells in the DMH is likely driven by increases in ΔFosB. The DMH is a region known to be involved in stress response and body weight regulation (DiMicco et al., 2002; DiMicco and Zaretsky, 2007), and increases in FosB/ΔFosB protein expression have been previously observed in the DMH following repeated restraint stress (Flak et al., 2012). More specifically, the DMH mediates the metabolic, but not the anorectic, responses to leptin (Rezai-Zadeh et al., 2014). This is consistent with our finding that chronic mT exposure does not attenuate weight gain through changes in food intake, and suggests that changes in DMH neural activity may influence body weight via a metabolic mechanism.

In summary, the current data add to the complex picture of predator threat response and weight regulation in prey mammals. We demonstrate that C57BL/6J laboratory mice will regulate their body weight in response to fear-inducing odor, but that this response, at least for mT, is likely not mediated through changes in food intake. Interestingly, we find that this weight regulation occurs regardless of dietary fat content, demonstrating the broad influence of innate threat in regulation of body weight even in the context of obesogenic diet. More study of this ecologically relevant and obesity-preventing neurophysiological pathway is important for gaining a broader picture of the biology of weight regulation.

## Author Contributions

EAG-B conceived, designed, and conducted experiments, analyzed data, interpreted results, and wrote the manuscript. JRT conducted experiments and edited manuscript. RJD helped conceive and design experiments, interpreted results, and revised manuscript. All authors approved the submitted version.

## Funding

National Science Foundation Graduate Research Fellowship to EAG-B.

## Conflict of Interest Statement

The authors declare that the research was conducted in the absence of any commercial or financial relationships that could be construed as a potential conflict of interest.
