# Supplementary material for: Innate Fear-Induced Weight Regulation in the C57BL/6J Mouse
Source: Front Behav Neurosci. 2016 Jul 4;10:132. doi: 10.3389/fnbeh.2016.00132 (PMC4930939; doi:10.3389/fnbeh.2016.00132)

Supplementary methods

Freezing in small chamber

Three cages (n=5 per cage) of 8 week old C57BL/6J mice were habituated to a small (12x7x7cm) plastic chamber with two 10-minute sessions over 2 days. On the 3rd day mice were exposed to 30µl of distilled water on a small piece of KimWipe placed on a small weigh boat ("scent dish") affixed to the right side of the chamber. Behavior was recorded using a camcorder. On the 4th day, liquid odorants (either 9.8 µl 50% mT; 52.8 µl BA; or 30 µl vanilla extract, neutral scent, McCormick, Sparks, MD) were pipetted onto the scent dish and behavior was again recorded over the 10-minute session. Freezing time (defined as time spent when the animal's only movements are breathing and whisker movements) was manually scored from randomized video files using the behavioral software AnyMaze.

Supplementary Figure legends

Supplementary Figure 1. mT robustly increases freezing behavior. Three groups of mice were exposed to 10-minute sessions of water (no scent) and then scent (mT, BA, or vanilla) in a small chamber and scored for freezing behavior. There was a highly significant effect of scent vs no scent (2-way ANOVA, $p<0.0001$, $F=32.69$), type of scent ($p<0.0001$, $F=32.61$), as well as a significant interaction ($p=0.0005$, $F=15.39$). Multiple comparisons tests showed a highly significant ($p<0.0001$) increase in freezing in response to mT compared to BA, neutral scent (vanilla), and no scent. No significant difference

was seen between BA and neutral scent, or between no-scent (water) and BA or neutral scent. All error bars shown represent SEM.

Supplementary Figure 2. mT attenuates weight gain in the repeated locomotor testing cohort. Mice exposed to 4 weeks of daily mT exposure during the repeated locomotor activity cohort gained significantly less weight than BA-exposed mice of the same cohort. All error bars shown represent SEM.

Supplementary Figure 3. Acute mT exposure does not alter *Ucp1* mRNA in brown adipose tissue. Uncoupling protein 1 (*Ucp1*) mRNA levels measured with qPCR from brown adipose tissue in mice exposed to mT or BA 5 hours before sacrifice. Fold change compared with TATA-binding protein and calculated using the $2^{-\Delta\Delta Ct}$ method. All error bars shown represent SEM.

Supplementary Figure 4. **Brain dissection locations for FosB/ΔFosB protein measurement.** Approximate locations and shape of regions dissected from brains of chow-fed mice exposed to 6 weeks of daily mT or BA scent. Brain images adapted from The Mouse Brain in Sterotaxic Coordinates, Paxinos and Franklin (Second Edition). All error bars shown represent SEM.

Supplementary Figure 1

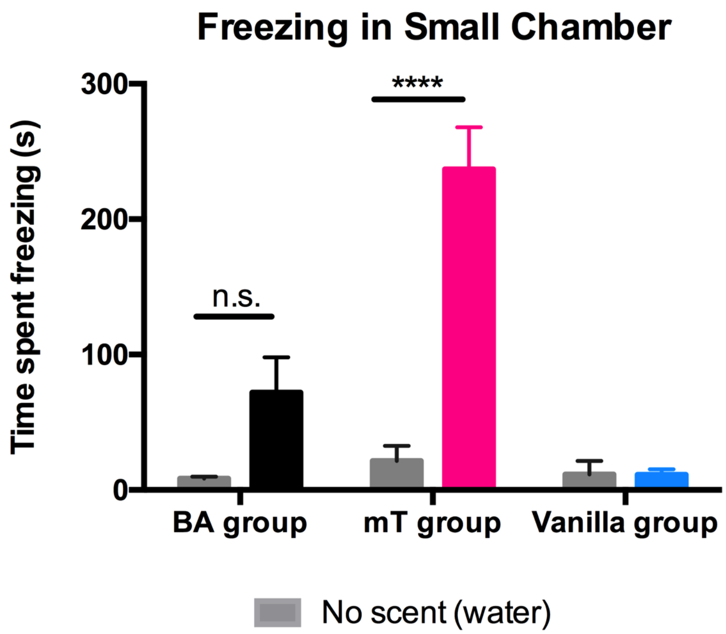

Supplementary Figure 2

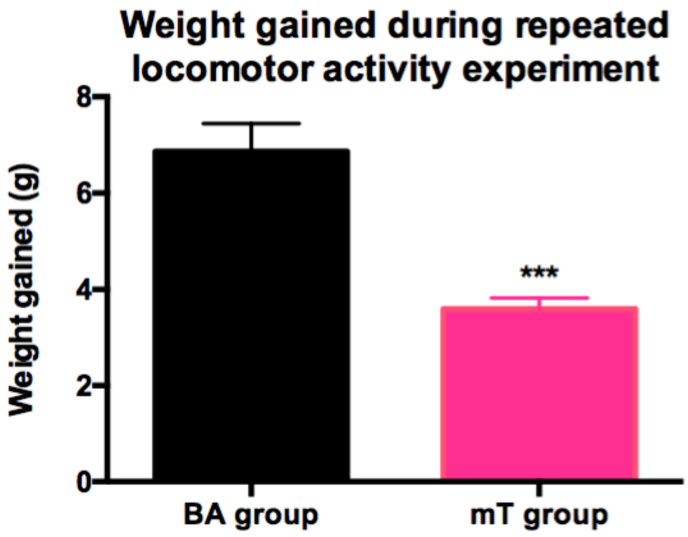

Supplementary Figure 3

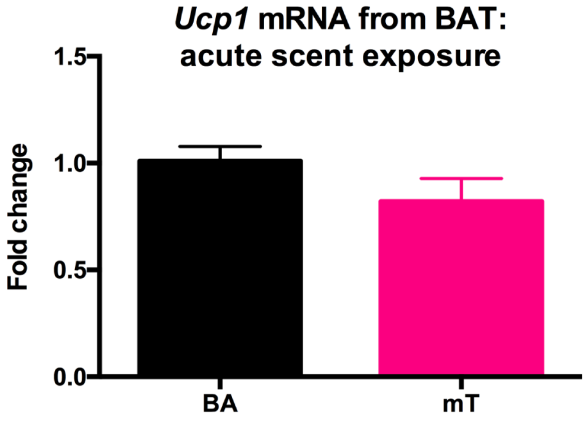

Supplementary Figure 4

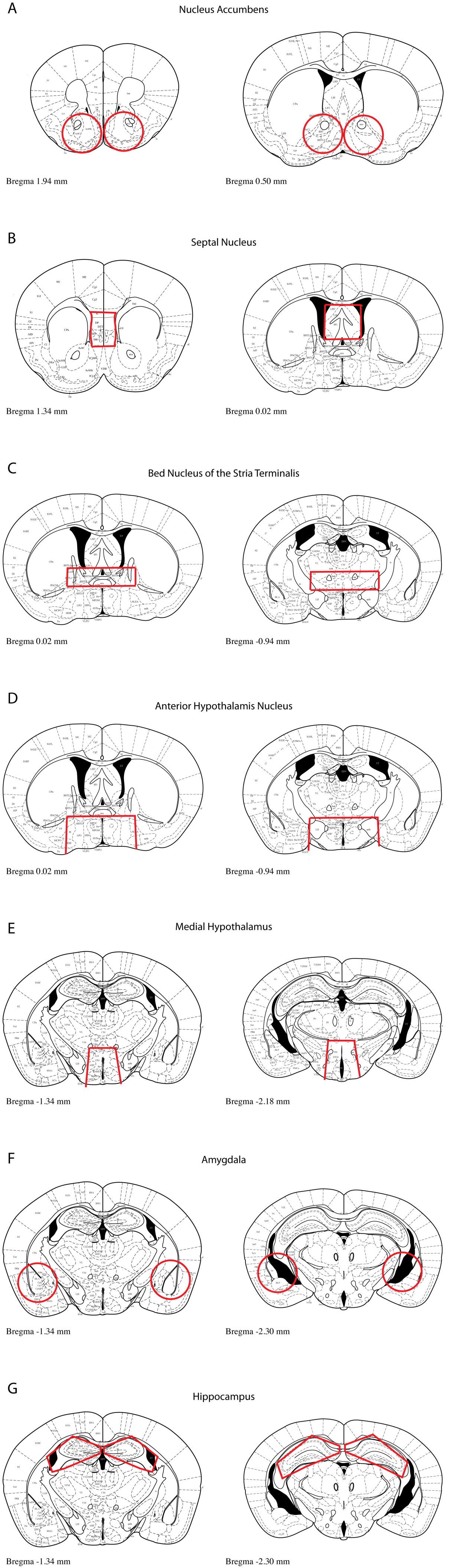

Supplement: Supplementary file 1 [file Image_1.pdf]
